# Comparative Efficacy of Two‐Session Radiofrequency Ablation Versus Transarterial Embolization Followed by Radiofrequency Ablation for the Treatment of Large Benign Thyroid Nodules

**DOI:** 10.1155/ije/5252455

**Published:** 2026-01-12

**Authors:** Cheng-Kang Wang, Yueh-Sheng Chen, Chen-Kai Chou, Wei-Chih Chen, Yuan-Pin Lin, An-Ni Lin, Chih-Ying Lee, Sheng-Dean Luo, Wei-Che Lin

**Affiliations:** ^1^ Department of Diagnostic Radiology, Kaohsiung Chang Gung Memorial Hospital and Chang Gung University College of Medicine, Kaohsiung, Taiwan, cgmh.org.tw; ^2^ Department of Diagnostic Radiology, Kaohsiung Municipal Fong Shan Hospital-Under the Management of Chang Gung Medical Foundation, Kaohsiung, Taiwan; ^3^ Department of Diagnostic Radiology, Kaohsiung Municipal Ta-Tung Hospital, Kaohsiung, Taiwan, kmtth.org.tw; ^4^ Thyroid Head and Neck Ablation Center, Kaohsiung Chang Gung Memorial Hospital, Kaohsiung, Taiwan, cgmh.org.tw; ^5^ School of Medicine and Doctoral Program of Clinical and Experimental Medicine, College of Medicine National Sun Yat-Sen University, Kaohsiung, Taiwan; ^6^ Division of Endocrinology and Metabolism, Department of Internal Medicine, Kaohsiung Chang Gung Memorial Hospital, and Chang Gung University College of Medicine, Kaohsiung, Taiwan; ^7^ Department of Otolaryngology, Kaohsiung Chang Gung Memorial Hospital, Chang Gung University College of Medicine, Kaohsiung, Taiwan, cgu.edu.tw; ^8^ Institute of Medical Science and Technology, National Sun Yat-sen University, Kaohsiung, Taiwan, nsysu.edu.tw; ^9^ Department of Electrical Engineering, National Sun Yat-sen University, Kaohsiung, Taiwan, nsysu.edu.tw

**Keywords:** large benign thyroid nodules, radiofrequency ablation, thyroid, transarterial embolization, ultrasound

## Abstract

**Purposes:**

To treat large benign thyroid nodules (BTNs), radiofrequency ablation (RFA) and transarterial embolization (TAE) combined with RFA are both used. This study aimed to compare the effectiveness of both treatments.

**Methods:**

Nineteen subjects with 20 BTNs received two sessions of RFA, while eight patients with 10 BTNs underwent TAE followed by RFA in a single medical center. Propensity score matching (PSM) was utilized to control for inherent potential biases by matching similar characteristics between the two groups.

**Results:**

Prior to treatment, a larger median nodule volume was observed in the TAE with the RFA group (150.75 ± 202.61 mL) compared to that of the double‐session RFA group (81.44 ± 58.00 mL). The volume reduction ratio (VRR) was found to be 74.60 ± 14.49% in the TAE/RFA group and 83.93 ± 12.70% in the double‐session RFA group. To account for follow‐up duration, we calculated the variation of VRR (ΔVRR, %) divided by the follow‐up time in days. The TAE with the RFA group showed a median reduction of 0.17 ± 0.05%/day, while the double‐session RFA group showed 0.13 ± 0.05%/day, with a *p* value of 0.040. The TAE with the RFA group exhibited a relatively higher daily VRR after treatment. Additionally, a higher nodular volume reduction was observed in the TAE with the RFA group due to the larger pretreatment nodular volume.

**Conclusions:**

Our findings suggest that TAE combined with RFA may be a more effective treatment option for large BTNs compared to two sessions of RFA alone, thus providing an alternative treatment approach.

**Key Points**

• Double‐session RFA and TAE are both therapeutic modalities for the treatment of large benign thyroid nodules.
• The group of TAE combined with RFA exhibited a relatively higher median daily VRR after treatment.
• TAE followed by RFA may be more effective for the treatment of large benign thyroid nodules than two sessions of RFA.

## 1. Introduction

Thyroid nodules are among the most common clinical findings in the general population, with prevalence estimates ranging from 16% to 67% depending on the detection methods and demographic characteristics of the population studied [1]. Their occurrence increases significantly with advancing age, female sex, and prior exposure to ionizing radiation [1]. In Taiwan, ultrasound‐based population studies have reported a prevalence of approximately 16%–19%, consistent with rates observed worldwide [1]. Although the majority of thyroid nodules (around 90%–95%) are benign, distinguishing benign from malignant nodules remains a crucial clinical challenge [1].

Benign thyroid nodules (BTNs) require intervention when they produce symptomatic compression such as airway narrowing, dysphagia, or neck discomfort, when cosmetic concerns adversely affect quality of life, when intrathoracic extension (intrathoracic goiter, ITG) causes functional limitation, or when progressive growth is documented [1, 2]. Surgery has long been considered the standard treatment for symptomatic BTNs, but it carries potential risks, including permanent hypoparathyroidism, recurrent laryngeal nerve injury, and general anesthesia‐related morbidity [1].

Management becomes more complex for large BTNs, typically defined as those with a volume > 30 mL or a maximal diameter > 4 cm. These nodules show higher rates of regrowth or incomplete treatment response compared with smaller nodules [1]. When extending into the mediastinum as ITGs, surgical management may require extracervical approaches such as manubriotomy or thoracotomy, further increasing procedural risks [2–4]. Additionally, a considerable proportion of patients decline surgery or are deemed medically ineligible due to advanced age or cardiopulmonary comorbidities [1, 5].

Several previous studies have reported on the efficacy of transarterial embolization (TAE) as applied in the treatment of nodular goiters, Graves’ disease, and hyperthyroidism [6–8]. Selective embolization of thyroid arteries has an outcome similar to a surgical subtotal thyroidectomy [9], while exhibiting a lower complication rate and an acceptable VRR as compared to RFA [6].

While the clinical effectiveness of both RFA and TAE in the treatment of BTNs has been demonstrated in separate studies, there is currently a lack of literature directly comparing the therapeutic efficacy of these two approaches when RFA is combined with TAE versus applied as multiple sessions alone [1]. This knowledge gap is particularly important for very large benign nodules, where achievement of satisfactory treatment response remains challenging and treatment failure rates are higher.

Therefore, we undertook this retrospective study to compare the effectiveness and safety of two treatment approaches for large BTNs: (1) two‐session RFA and (2) combined TAE followed by RFA. We aimed to evaluate the comparative therapeutic efficacy, safety profiles, and patient outcomes between these two treatment modalities in patients with large BTNs who were unsuitable for or refused surgical intervention.

## 2. Patients and Methods

The institutional review board of the Chang Gung Memorial Hospital approved this retrospective study, and informed consent was obtained from all patients prior to the RFA and TAE procedures. All subjects received two separate fine‐needle aspiration (FNA) or core‐needle biopsy (CNB) procedures in different parts of the thyroid lesions to confirm the benign nature of the thyroid nodules. There were no suspicious malignant features on US examination, including hypoechoic taller‐than‐wide shape, or ill‐defined margins. Two radiologists experienced in the thyroid RFA field reviewed the images.

The inclusion criteria for this study were as follows: (1) a large thyroid nodule volume (> 30 mL); (2) a maximum diameter of the thyroid nodule > 4 cm; (3) serum thyroid hormone, thyroid‐stimulating hormone (TSH), and thyroglobulin levels within normal ranges; (4) follow‐up period ≥ 6 months after RFA or TAE; and (5) patients who refused surgery. The exclusion criteria were as follows: (1) biopsy results showing follicular neoplasm or malignancy; (2) suspicious malignant features on ultrasonography even with a benign biopsy result; (3) coagulation disorder, contrast medium allergy, or other contraindications of angiography. Due to the relatively high risk of recurrence or regrowth, and intrathoracic extension, we recommended patients undergo two sessions of RFA or TAE procedure combined with RFA to downgrade the level of BTN during the initial RFA or TAE procedure. We then ablated the majority of BTNs in the subsequent RFA to achieve a better VRR. All patients were informed as to the risks and benefits associated with the RFA, TAE, and surgical procedures. A majority of patients refused surgery, while others were not suitable surgical candidates due to the underlying disease. There was a shared decision‐making process, which was well documented during which time patients were encouraged to sufficiently deliberate and express their opinions during the discussions.

A total of 27 patients presenting with 30 BTNs who underwent procedures at our institution between March 2017 and March 2021 were retrospectively enrolled in this study. Among them, 19 patients with 20 nodules who underwent two sessions of RFA at separate times were included in Group 1, and 8 patients with 10 nodules who received TAE with subsequent RFA were in Group 2.

### 2.1. Pretreatment Assessment

All the patients underwent the pretreatment assessments at the outpatient service of the departments of endocrinology, otolaryngology, thyroid interventional radiology, or surgery. The thyroid function tests included triiodothyronine (T3), free thyroxine (fT4), and TSH. In addition, US study combined with computed tomography (CT), or magnetic resonance imaging (MRI) was used to determine the size, echogenicity, margin, shape, component, calcification, location, and the level of intrathoracic extension. The nodular volume was calculated using the following equation: *V* = *πabc*/6 (*V*: volume; *a*: the largest diameter; *b* and *c*: the other double perpendicular diameters).

### 2.2. Radiofrequency Ablation

Prior to the ablation procedures, patients in the RFA group were informed of the requirement for two sessions of RFA to achieve a better VRR due to the relatively large nodular volume. The ablation procedures were performed by a radiologist with over 15 years of experience in US‐guided procedures. Each RFA procedure was performed with an RF generator (VIVA, STARmed and M2004, RF Medical) with an internally cooled electrode (18 gauge, with 10, 7, or 5‐mm active tip), the size of which was based on the nodular size and the status of the surrounding critical structures. The RFA procedures were performed using a trans‐isthmic approach, with hydrodissection and the moving‐shot technique. When all visual fields of the nodule had changed to transient hyperechoic zones, the ablation procedure was terminated.

### 2.3. TAE Combined With RFA

All TAE procedures were performed using the right femoral arterial approach with a 5‐F sheath. A catheter was advanced over a hydrophilic guide wire, and the nodular‐side superior and inferior thyroid arteries were selectively catheterized, identifying the thyroid arteries that fed the nodule (s). After a 2.7‐F microcatheter was selectively advanced into the major branches of the thyroid arteries and placed in a secure position, the artery was embolized with 300–500‐μm Embosphere microspheres until a decreased blood flow was observed. To ensure optimal embolization, the proximal part of the feeding arteries was occluded with coils. Based on the angiography results, embolization of the nodular‐side superior and inferior thyroid arteries was performed. Preservation of one inferior thyroid artery was considered to avoid the potential risk of hypoparathyroidism if a patient presented with bilateral thyroid nodules. The procedure was considered completed when the target arteries were optimally embolized. Some patients received subsequent RFA on the same day, while others received RFA several months later, depending on the level of intrathoracic extension and nodular components. After the TAE procedure, patients were observed overnight to monitor for potential complications, including airway compression and thyroid storm.

### 2.4. Follow‐Up Evaluations

Post‐treatment follow‐up US at 1, 3, 6, and 12 months were performed to evaluate the VRR. The VRR was assessed by medical imaging and was calculated by the following equation: volume reduction ratio (%) = initial volume (mL) − final volume (mL) × 100/initial volume. The vascularity of the ablated nodules, and cosmetic score and symptomatic scores were also evaluated at each follow‐up. A follow‐up contrast‐enhanced CT or MRI examination was performed at 6 months after the procedure to establish the thyroid gland volume. The serum levels of T3, fT4, and TSH were measured prior to the procedures and at 6 months. To evaluate the different treatment outcomes by considering the time factor, the imaging follow‐up interval between the RFA or TAE procedure and CT or MRI imaging study is represented as Δtime.

### 2.5. Analyses and Statistics

Statistical analyses were performed by using SPSS, version 26 (SPSS, Inc., Chicago, IL, USA). All data are given as the mean ± standard deviation (SD). Demographic characteristics, ultrasound, CT, or MRI results between the double groups were compared. Standard chi‐squared and Fisher’s exact tests were used for group comparisons of the categorical data. Group comparisons for the continuous variable data were performed by using the Mann–Whitney *U* test and the Kruskal–Wallis test (SPSS, Inc., Chicago, IL, USA).

We used R (R Core Team, 2014) to demonstrate propensity score matching (PSM) and propensity score weighting (PSW) in a regression model. To control for inherent potential biases, the one‐to‐one PSM by using the nearest‐neighbor matching method was applied to the two groups based on age, sex, largest diameter, volume, and follow‐up period. PSW with inverse probability of treatment weighting (IPTW) and standardized mortality ratio weighting (SMRW) were also performed to control for the influence of participants by weighting their responses based on their propensity scores. Average treatment effect on the treated (ATT) is an evaluator of PSW, which is the difference in the outcome variable between the average score for the two groups. Patient characteristics and outcomes were compared between the two groups both before and after matching. A 2‐sided *p* < 0.05 was considered as statistically significant.

## 3. Results

The demographic data of the two groups are shown in Table 1. There were 19 subjects with 20 BTNs included in the group of double‐session RFA group and 8 patients with 10 BTNs included in the TAE with the RFA group. There were no differences in age and sex distribution between the two groups. The thyroid functions between the two groups were within normal ranges.

**Table 1:** Demographic data of double‐session RFA patients and TAE with RFA patients.

	**Double-session RFA**	**TAE with RFA**	**p** **value**

Subjects	19	8	
Nodules	20	10	
Gender (F:M)	16:3	7:1	0.455
Age	42.20 ± 9.29	47.00 ± 12.57	0.859
T3 (ng/dL) pre	103.78 ± 22.86	107.47 ± 64.96	0.055
Free T4 (μg/dL) pre	1.19 ± 0.14	1.21 ± 0.49	0.413
TSH (μIU/mL) pre	1.16 ± 0.65	0.79 ± 0.52	0.508
Prevolume (mL)	81.44 ± 58.00	150.75 ± 202.61	0.026
Postvolume 1^st^ RFA (mL)	39.77 ± 40.42		
Postvolume^total^ (mL)	14.13 ± 13.89	32.35 ± 36.64	0.010
VRR (%) post 1^st^ RFA	54.07 ± 19.50		
VRR (%)^total^	83.93 ± 12.70	74.60 ± 14.49	0.413
Δtime (days)	170.60 ± 149.70		
Δtime^total^ (days)	733.80 ± 319.54	446.80 ± 87.26	0.001
ΔVRR/Δtime^total^ (%/days)	0.13 ± 0.05	0.17 ± 0.05	0.040
T3 (ng/dL) post	88.07 ± 12.78	87.68 ± 11.45	0.392
Free T4 (μg/dL) post	1.10 ± 0.23	0.99 ± 0.23	0.705
TSH (μIU/mL) post	1.70 ± 0.87	1.42 ± 0.78	0.997
kcal/mL	0.30 ± 0.14	0.24 ± 0.18	0.773
J/mL	1271.11 ± 611.34	997.04 ± 768.45	0.773

*Note:* Δtime: interval between the first RFA and postinitial RFA CT or MRI examination. Δtime^total^: interval between the date of the first RFA and the date of postsecond RFA CT or MRI examination or interval of the initial time follow‐up CT or MRI examination after post‐TAE RFA.

### 3.1. Group 1: Double‐Session RFA

The double‐session RFA group data are shown in Tables 1 and 2. The mean pretreatment nodular volume was 81.44 ± 58.00 mL, while the average residual volume of the thyroid nodules was about 39.77 ± 40.42 mL. The mean VRR was 54.07 ± 19.50% after the first RFA. The mean interval between the first and the second RFA was 347.55 ± 206.24 days (ranging from 111 to 881 days). After the second RFA procedure, the median residual volume of the ablated nodules was 14.13 ± 13.89 mL, while VRR was 83.93 ± 12.70%. The medical imaging interval was 733.80 ± 319.54 days between the two RFA sessions. The mean daily VRR was 0.13 ± 0.05%/day, indicating an approximately 0.13% daily volume reduction ratio after the ablation treatment.

**Table 2:** Patient characteristics of the double‐session RFA group.

Patient no.	Age	Gender	Thyroid nodules	Prevolume	Intrathoracic extension	Time between double RFA in days
1	43	F	Predominantly solid	56.91	Grade 1 Type A	374
2	45	F	Predominantly solid	30.13	No	296
3	25	F	Predominantly solid	72.06	No	217
4	37	F	Predominantly solid	148.96	Grade 2 Type B	112
5	38	F	Solid	30.01	Grade 1 Type A	497
6	35	F	Solid	68.21	Grade 2 Type B	365
7–1	42	F	Predominantly solid (right)	39.86	No	304
7–2	42	F	Predominantly solid (left)	30.06	No	119
8	66	M	Predominantly solid	251.98	Grade 2	191
9	37	F	Solid	173.27	Grade 2 Type B	210
10	38	F	Predominantly solid	101.82	Grade 2 Type B	383
11	58	F	Solid	55.41	Grade 3 Type C	142
12	47	M	Predominantly solid	76.69	Grade 1 Type A	896
13	44	F	Solid	138.45	Grade 1 Type B	222
14	41	F	Solid	79.52	Grade 1 Type A	209
15	42	F	Solid	47.4	Grade 1 Type A	828
16	54	M	Solid	37.25	Grade 1 Type A	479
17	39	F	Predominantly solid	53.65	No	534
18	43	F	Solid	82.87	Grade 1 Type A	317
19	28	F	Solid	80.73	Grade 1 Type A	289

*Note:* The nodules were classified into three grades and three types using the CT cross‐sectional imaging classification system. Classification into the craniocaudal plane is based on the lower margin of the thyroid, as follows: Grade 1 (the inferior margin is between the thoracic inlet and the aortic arch convexity); Grade 2 (the inferior margin is between the aortic arch convexity and concavity); and Grade 3 (the inferior margin is below the aortic arch concavity). Classification in the anteroposterior plane is based on the relation of the main mass of the thyroid gland and the aortic arch or its branches and the trachea, as follows: Type A (prevascular); Type B (retrovascular‐paratracheal); and Type C (retrotracheal).

### 3.2. Group 2: TAE With RFA

The data for the TAE combined with the RFA group are shown in Tables 1 and 3. The median pretreatment nodular volume was 150.75 ± 202.61 mL, and the residual volume of the thyroid nodules was 32.35 ± 36.64 mL. The median VRR was 74.60 ± 14.49%. The median interval between the TAE and RFA procedures was 81.30 ± 95.29 days (ranging from 0 to 207 days). Significantly larger residual thyroid lesions were noted in the TAE with the RFA group as compared to that of the double‐session RFA group. The median medical imaging interval is 446.80 ± 87.26 days between the pretreatment assessment and the completed TAE with RFA treatments. The median daily VRR was 0.17 ± 0.05%/day. After treatment, thyroid functions were within normal range in both groups, with no significant difference. The energy delivered per nodular volume is considered a major factor affecting the VRR after RFA [5]. As such, the median RFA energy delivered was 1271.11 ± 611.34 J/mL (0.30 ± 0.14 kcal/mL) in the double‐session RFA group and was 997.04 ± 768.45 J/mL (0.24 ± 0.18 kcal/mL) in the TAE with the RFA group, indicating no significant difference in terms of RFA energy delivered between the two groups.

**Table 3:** Patient characteristics of the TAE with the RFA group.

Patient no.	Age	Gender	Thyroid nodules	Prevolume	Intrathoracic extension	Time between TAE and RFA in days
1	39	M	Predominantly cystic	698.39	Grade 1 Type A	0
2	81	F	Solid	158.69	Grade 2 Type C	1
3	37	F	Solid	30.12	Grade 1 Type A	0
4	43	F	Solid	87.13	Grade 1 Type A	0
5	46	F	Predominantly solid	34.82	Grade 1 Type A	0
6	38	F	Predominantly solid	39.13	No	122
7	45	F	Predominantly solid	223.75	Grade 1 Type A	73
8–1	47	F	Predominantly solid (isthmus)	129.91	Grade 1 Type A	207
8–2	47	F	Solid (right)	55.91	No	207
8–3	47	F	Solid (left)	50.13	No	207

*Note:* The nodules were classified into three grades and three types using the CT cross‐sectional imaging classification system.

There were no major complications in both groups, such as permanent voice change, delayed rupture, or stroke. The most common complication among patients in both groups was pain and swelling, which were relieved within 1 week of the procedures.

## 4. PSM

To control for potential biases, propensity scores were used as weights to account for selection assignment differences between the two groups. Several variates, including the number of subjects, age, pre‐ and post‐treatment volumes, VRR, ΔVRR/Δtime, and J/mL, were analyzed in PSM and PSW. The one‐to‐one PSM by using the nearest‐neighbor matching method was applied with calipers of 0.2, 0.3, and 2.5, while the number of subjects was 8, 9, and 10, respectively, as shown in Supporting Table 1. The two different methods of PSW, IPTW, and SMRW are shown in Supporting Table 2. After the IPTW analysis, the number of subjects was 29.72 in the double‐session RFA group and 29.96 in the TAE with the RFA group. And after SMRW analysis, the number of subjects was 20.00 in the double‐session RFA group and 19.96 in the TAE with the RFA group. The median VRR together with the time factor was more notable in the TAE with the RFA group than the double‐session RFA group, although there was no significant difference in all variates after applying PSM and PSW analyses.

## 5. Discussion

The present study of patients with large BTNs treated by two RFA sessions or TAE combined with RFA demonstrates that both therapeutic modalities achieved a satisfactory VRR, with a mean volume reduction of 74% in the TAE with the RFA group and 83% in the double‐session RFA group. The preprocedural assessment revealed a larger average nodular volume in the TAE group as compared to that in the double‐session RFA group; hence, a relatively higher nodular volume reduction in the TAE group would be expected, accompanied by a lower VRR as compared to that in the double‐session RFA group. According to previous studies, a single session of RFA is less effective for BTNs with a volume of > 30 mL than for smaller BTNs [1]. Thus, for patients with large BTNs who refuse surgery, a single session of RFA may not be expected to achieve an acceptable VRR, suggesting that TAE would be a more effective treatment.

Multiple sessions of RFA can be effective in reducing the volume of large BTNs. In our study, the shortest interval between RFA sessions was 6 months, as the effect of the initial RFA will generally persist for at least this amount of time. In cases of the maximum diameter of a large BTN is > 5 cm and with a volume > 30 mL, more than a single session of RFA should be considered to achieve a satisfactory VRR. In this regard, a down‐staging procedure is a valuable step in a multisession RFA. For ITGs, the initial RFA can act to reduce the ITG grade, facilitating subsequent RFA procedures to achieve a better VRR. As there is a higher complication rate among large BTNs [1], multiple sessions of RFA are a strategy applied to reduce the risk of voice change, delayed rupture, and other complications.

**Figure 1:**
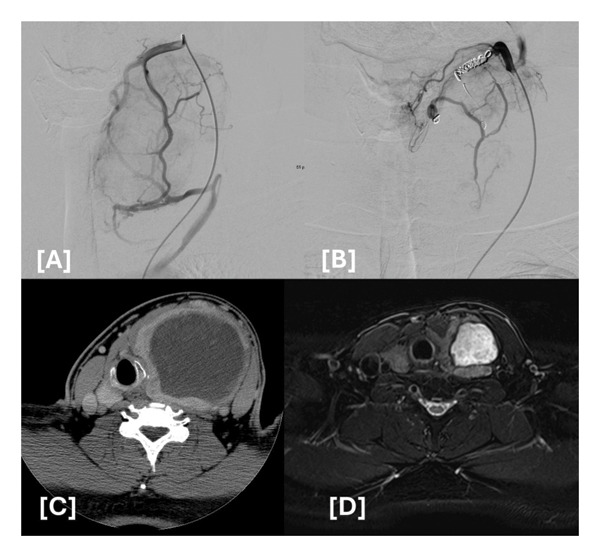
Patient one of the TAE with the RFA group. (A) Diagnostic angiography before embolization. (B) Post‐TAE with embosphere followed by coil embolization. (C) CT study before TAE. (D) MRI study at a 6‐month follow‐up post TAE with RFA.

TAE combined with RFA was used to treat patients presenting with large BTNs in this study. Among this group of patients, the average pretreatment nodular volume was significantly higher than that of the double‐session RFA group. Previous studies have indicated that TAE is a more effective treatment modality than traditional thermal ablation for large nodules [6, 10, 11]. In our study, 5 patients underwent TAE and RFA in the same day, while these BTNs were defined as mixed solid and cyst, or hemorrhagic cysts. To effectively decrease vascularity, TAE was performed with 300–500 μm Embosphere microspheres until optimal stasis was achieved followed by coils. For BTNs with a cystic component, TAE was introduced initially to control vascularity and possibly prevent hemorrhage during the ablation procedure, while the subsequent RFA procedure could focus on the solid section of the nodule (Figure 1). The other three patients received the two procedures with an interval of between 2 and 6 months. Of these patients, two had predominantly solid thyroid nodules, while one patient presented with three large predominantly solid nodules. These patients underwent the RFA procedure after a period of time to avoid postembolization compression of the trachea due to a large solid nodule component. Of note, TAE acts as a downgrading procedure for large BTNs. The initial TAE procedure serves to embolize the inferior thyroid arteries, which supply the intrathoracic part of large BTNs, allowing for the subsequent RFA procedure to effectively ablate the residual sections of BTNs.

In our procedural analysis, we used the PSM by matching each treated unit with a nontreated unit with similar characteristics among the two groups. Among three different caliper distances, there was no significant difference in age and pretreatment volume between the two groups; however, the TAE with the RFA group exhibited a relatively larger pretreatment volume. Additionally, IPTW and SMRW using the propensity score apply weights based on the propensity score to create a synthetic sample in which the distribution of measured baseline covariates is independent of treatment assignment. Although there remained no significant difference in terms of post‐treatment volume, VRR, and ΔVRR/Δtime, several trends were revealed in the results. More specifically, we noted a higher absolute reduced nodular volume in the TAE with the RFA group than the double‐session RFA groups due to the larger pretreatment nodular volume. The TAE with the RFA group also exhibited a relatively higher median daily VRR after treatment. These results indicate that TAE or TAE combined with RFA may be more effective than multiple sessions of RFA to treat large BTNs.

There are several limitations to this study. First, as this was a retrospective single‐center study, uncontrolled bias could have been introduced. Further prospective study or randomized controlled study is required to validate our results. Second, the sample size of the two groups was too small, although PSM was used to mimic some of the characteristics of a randomized controlled trial. Third, the long‐term outcomes of the two groups should be determined by continued follow‐up. Finally, a separate study should be conducted to compare the effectiveness of the TAE combined with RFA treatment with that of pure TAE treatment.

In conclusion, our study indicates that TAE combined with RFA showed a potential benefit in volume reduction for large BTNs compared to two‐session RFA, though further studies are required to confirm this finding. As an initial down‐staging procedure, TAE can act to effectively reduce a greater nodular volume without the risk of major complications. The findings may assist physicians to better inform the decision‐making process when discussing the various treatment options available to patients presenting with large BTNs.

**Nomenclature**
US: Ultrasound
RFA: Radiofrequency ablation
TAE: Transarterial embolization
BTNs: Benign thyroid nodules
VRR: Volume reduction ratio
ITG: Intrathoracic goiter
FNA: Fine‐needle aspiration
CNB: Core‐needle biopsy
T3: Triiodothyronine
fT4: free thyroxine
TSH: Thyroid‐stimulating hormone
CT: Computed tomography
MRI: Magnetic resonance imaging
PSM: Propensity score matching
PSW: Propensity score weighting
IPTW: Inverse probability of treatment weighting
SMRW: Standardized mortality ratio weighting
ATT: Average treatment effect on the treated

## Conflicts of Interest

The authors declare no conflicts of interest.

## Funding

No funding was received for this manuscript.

## Supporting Information

Supporting Table 1: Clinical characteristics and treatment factors of the two groups with propensity score matching (PSM) (1:1).

Supporting Table 2: Clinical characteristics and treatment factors of the two groups with propensity score weighting.

IPTW: inverse probability of treatment weighting.

SMRW: standardized mortality ratio (SMR) weighting.

## Supporting information


**Supporting Information** Additional supporting information can be found online in the Supporting Information section.

## Data Availability

The data that support the findings of this study are available on request from the corresponding author. The data are not publicly available due to privacy or ethical restrictions.
