# Supplementary material for: Comparative Efficacy of Two‐Session Radiofrequency Ablation Versus Transarterial Embolization Followed by Radiofrequency Ablation for the Treatment of Large Benign Thyroid Nodules
Source: Int J Endocrinol. 2026 Jan 12;2026:5252455. doi: 10.1155/ije/5252455 (PMC12793772; doi:10.1155/ije/5252455)
Supplement: Supplementary file 1 — Supporting Information Additional supporting information can be found online in the Supporting Information section. [file IJE-2026-5252455-s001.docx]

**Table S1.** Clinical characteristics and treatment factors of the two groups with propensity score matching (PSM) (1:1)

| **Characteristics** | | | N | Age | Pre-volume | Post-volume (mL) ^total^ | VRR (%) ^total^ | ΔVRR/Δtime ^total^(%/days) | J/mL |
| --- | --- | --- | --- | --- | --- | --- | --- | --- | --- |
| Before PSM | | RFA twice | 20 | 42.20±9.29 | 81.44±58.00 | 14.13 ± 13.89 | 83.93 ± 12.70 | 0.13 ± 0.05 | 1271.11 ± 611.34 |
|  |  | TAE with RFA | 10 | 47.00±12.57 | 150.75±202.61 | 32.35 ± 36.64 | 74.60 ± 14.49 | 0.17 ± 0.05 | 997.04 ± 768.45 |
|  |  | *p* value |  | 0.43 | 0.46 | 0.01 | 0.41 | 0.04 | 0.77 |
| After PSM | Caliper=0.2 | RFA twice | 8 | 42.88±7.28 | 84.14±41.48 | 24.98 ± 19.98 | 79.62 ± 14.85 | 0.15 ± 0.12 | 912.64 ± 574.39 |
|  |  | TAE with RFA | 8 | 43.75±4.10 | 91.30±66.45 | 26.73 ± 23.07 | 77.29 ± 16.13 | 0.16 ± 0.13 | 868.11 ± 691.95 |
|  |  | *p* value |  | 0.14 | 0.06 | 0.66 | 0.85 | 0.15 | 0.96 |
|  | Caliper=0.3 | RFA twice | 9 | 45.44±10.28 | 95.79±68.08 | 26.14 ± 20.39 | 80.39 ±13.35 | 0.16 ± 0.08 | 993.10 ± 697.38 |
|  |  | TAE with RFA | 9 | 47.89±12.99 | 102.90±67.30 | 28.40 ± 21.62 | 76.17 ±15.29 | 0.17 ± 0.11 | 939.76 ± 710.84 |
|  |  | *p* value |  | 0.20 | 0.19 | 0.67 | 0.53 | 0.16 | 0.96 |
|  | Caliper=2.5 | RFA twice | 10 | 46.30±10.07 | 96.24±67.45 | 28.27 ± 15.02 | 81.37 ± 15.03 | 0.16 ± 0.03 | 1293.72 ± 585.30 |
|  |  | TAE with RFA | 10 | 47.00±12.57 | 150.75±202.61 | 32.35 ± 36.64 | 74.60 ± 14.49 | 0.17 ± 0.05 | 997.04 ± 768.45 |
|  |  | *p* value |  | 0.06 | 0.36 | 0.36 | 0.46 | 0.13 | 0.58 |

**Table S2**. Clinical characteristics and treatment factors of the two groups with propensity score weighting

| **Characteristics** | | | N | Age | Volume | Post volume (mL) ^total^ | VRR (%) ^total^ | ΔVRR/Δtime ^total^(%/days) | J/mL |
| --- | --- | --- | --- | --- | --- | --- | --- | --- | --- |
| Before weight | | RFA twice | 20 | 42.20±9.29 | 81.44±58.00 | 14.13 ± 13.89 | 83.93 ± 12.70 | 0.13 ± 0.05 | 1271.11 ± 611.34 |
|  |  | TAE with RFA | 10 | 47.00±12.57 | 150.75±202.61 | 32.35 ± 36.64 | 74.60 ± 14.49 | 0.17 ± 0.05 | 997.04 ± 768.45 |
|  |  | *p* value |  | 0.43 | 0.46 | 0.01 | 0.41 | 0.04 | 0.77 |
| After weight | IPTW | RFA twice | 29.72 | 44.15±10.69 | 93.08±70.34 | 24.66 ± 22.10 | 78.06 ± 13.89 | 0.15 ± 0.07 | 1119.54 ± 756.72 |
|  |  | TAE with RFA | 29.96 | 44.59±9.39 | 98.62±141.44 | 25.47 ± 26.98 | 76.56 ± 14.97 | 0.16 ± 0.03 | 1098.76 ± 764.01 |
|  |  | *p* value |  | 0.91 | 0.05 | 0.96 | 0.94 | 0.09 | 0.96 |
|  | SMRW | RFA twice | 20.00 | 42.20±9.29 | 81.44±58.00 | 24.16 ± 23.52 | 78.37 ± 13.23 | 0.14 ± 0.09 | 1103.47 ± 749.98 |
|  |  | TAE with RFA | 19.96 | 43.39±6.96 | 72.50±84.79 | 23.47 ±24.98 | 74.98 ± 14.18 | 0.16 ± 0.04 | 1073.75 ± 781.63 |
|  |  | *p* value |  | 0.84 | 0.12 | 0.99 | 0.89 | 0.23 | 0.94 |

IPTW: inverse probability of treatment weighting

SMRW: standardized mortality ratio (SMR) weighting
